# Effects of Uni- vs. Bilateral Upper Limb Robot-Assisted Rehabilitation on Motor Function, Activities of Daily Living, and Electromyography in Hemiplegic Stroke: A Single-Blinded Three-Arm Randomized Controlled Trial

**DOI:** 10.3390/jcm12082950

**Published:** 2023-04-18

**Authors:** Runping Yuan, Xu Qiao, Congzhi Tang, Ting Zhou, Wenli Chen, Ruyan Song, Yong Jiang, Jan D. Reinhardt, Hongxing Wang

**Affiliations:** 1 Graduate School of Bengbu Medical College, Bengbu 233030, China; 2 Southeast University Zhongda Hospital, Nanjing 210003, China; 3 Institute for Disaster Management and Reconstruction of Sichuan University and Hong Kong Polytechnic University, Sichuan University, Chengdu 610041, China; 4 West China School of Public Health and West China Fourth Hospital, Sichuan University, Chengdu 610041, China; 5 The First Affiliated Hospital of Bengbu Medical College, Bengbu 233099, China; 6 Swiss Paraplegic Research, 6207 Nottwil, Switzerland; 7 Department of Health Sciences and Medicine, University of Lucerne, 6002 Lucerne, Switzerland; 8 Center for Rehabilitation Medicine, Jiangsu Province Hospital, First Affiliated Hospital of Nanjing Medical University, Nanjing 210029, China

**Keywords:** motor evoked potential, motor function, stroke, surface electromyography, upper limb robot-assisted rehabilitation

## Abstract

Objective: To evaluate if bilateral or unilateral upper limb robot-assisted rehabilitation training using a new three-dimensional end-effector robot that targets shoulder and elbow flexion and abduction is superior to conventional therapy with regard to upper extremity motor function recovery and neuromuscular improvement in stroke patients. Design: Randomized, controlled, parallel, assessor-blinded, three-arm clinical trial. Setting: Southeast University Zhongda Hospital Nanjing, Jiangsu, China. Methods: Seventy patients with hemiplegic stroke were randomly assigned to conventional training (Control, n = 23) or unilateral (URT, n = 23), or bilateral robotic training (BRT, n = 24). The conventional group received routine rehabilitation, 60 min/day, 6 days/week, for 3 weeks. For URT and BRT upper limb robot-assisted rehabilitation training was added. This was 60 min/day, 6 days/week, for 3 weeks. The primary outcome was upper limb motor function assessed with Fugl-Meyer–Upper Extremity Scale (FMA–UE). Secondary outcomes were activities of daily living (ADL) assessed with the Modified Barthel Index (MBI), Motor Evoked Potential (MEP) to assess corticospinal tract connectivity, Root Mean Square (RMS) value, and integrate Electromyography (iEMG) value recorded by surface electromyography to evaluate muscle contraction function. Results: The primary outcome indicator FMA–UE (least square mean (LSMEAN): 31.40, 95% confidence interval (95% CI): 27.74–35.07) and the secondary outcome indicator MBI (LSMEAN: 69.95, 95% CI: 66.69–73.21) were significantly improved in BRT as opposed to control (FMA–UE, LSMEAN: 24.79, 95% CI: 22.23–27.35; MBI, LSMEAN: 62.75, 95% CI: 59.42–66.09); and unilateral (FMA–UE, LSMEAN: 25.97, 95% CI: 23.57–28.36; MBI, LSMEAN: 64.34, 95% CI: 61.01–67.68). BRT also showed greater improvement in the anterior deltoid bundle with regard to muscle contraction function indicated by RMS (LSMEAN: 257.79, 95% CI: 211.45–304.12) and iEMG (LSMEAN: 202.01, 95% CI: 167.09–236.94), as compared to the controls (RMS, LSMEAN: 170.77, 95% CI: 148.97–192.58; iEMG, LSMEAN: 132.09, 95% CI: 114.51–149.68), and URT (RMS, LSMEAN: 179.05, 95% CI: 156.03–202.07; iEMG, LSMEAN: 130.38, 95% CI: 107.50–153.26). There was no statistically significant difference between URT and conventional training for any outcome. There was no significant difference in MEP extraction rate after treatment between groups (*p* = 0.54 for URT, *p* = 0.08 for BRT). Conclusions: A 60 min daily training for upper extremities using a three-dimensional end-effector targeting elbow and shoulder adding conventional rehabilitation appears to promote upper limb function and ADL in stroke patients only if delivered bilaterally. URT does not seem to result in better outcomes than conventional rehabilitation. Electrophysiological results suggest that training using a bilateral upper limb robot increases the recruitment of motor neurons rather than improving the conduction function of the corticospinal tract.

## 1. Introduction

Stroke is the second leading cause of both disability and mortality worldwide, with the highest burden of the disease falling on low- and middle-income countries [1]. Eighty percent of stroke survivors suffer from upper limb motor impairment. Of those, only one-third can recover practical function [2] and improvement of upper limb function is thus a major focus of rehabilitation after stroke [3]. Conventional rehabilitation treatments include occupational therapy; mirror therapy [4,5,6]; constraint-induced movement therapy [7,8]; and other active and passive training. These treatment options are, however, time-consuming, and resource-intensive, and outcomes often depend on the skills of the medical staff administering the treatments. Upper limb robot-assisted rehabilitation may have the potential to address these limitations.

Upper limb robot-assisted training is a promising developing post-stroke rehabilitation method with high-intensity, repetitive, task-oriented training characteristics [5,9]. Using a variety of games and real-time feedback it can also improve patient motivation [10,11]. In clinical practice, robotic training is used on the paretic arm only, or both arms and is therefore referred to as unilateral robot-assisted therapy (URT) or bilateral robot-assisted therapy (BRT), respectively.

URT aims to train the hemiplegic arm through repeated active or passive exercise. However, results regarding the effectiveness of URT remain inconclusive. In a single-blind, randomized controlled trial (RCT), Dehem et al. showed that dexterity and control of patients’ hands were improved to a greater degree after unilateral robotic-assisted therapy [5]. Similarly, Iwamoto et al. demonstrated in a single-center, randomized controlled trial that recovery of upper limb motor functions and activities of daily living (ADL) were increased when a unilateral upper limb robots-assisted was used in combination with occupational therapy, as compared to occupational therapy only [12]. Conversely, Rodgers et al. found that unilateral robotic-assisted therapy did not enhance upper limb function as compared to the usual care group [2]. Likewise, Takebayashi et al. reported that unilateral robotic self-training had no significant effects as opposed to non-robotic assisted self-training, but may improve upper-limb function when combined with outpatient rehabilitation [13].

BRT seems a viable alternative as it guides patients to complete symmetrical movements with the paralyzed limb using the motor information from the non-paralyzed side. We performed a systematic literature search (Supplementary Table S1) and found relatively few clinical trials studying the effects of bilateral upper limb robotic-assisted training. Of those trials, most used end-effector devices, and all of them Bi-Manu-Track assisted robots [14], of which the main function is to enable forearm pronation and supination and wrist flexion and extension. Hesse et al. reported the advantages of bilateral assisted robotic training in improving upper limb motor control and strength when they compared the Bi-Manu-Track bilateral assisted robotic training device with muscle electrical stimulation training of paralyzed wrist extensors [14]. Liao et al. found that symmetrical and bilateral robotic exercises combined with functional task training significantly improved motor function in patients with post-acute stroke as well as arm movement and self-perceived bilateral arm capacity [15]. Hsieh et al. found that a combination of task-oriented training and bilateral upper extremity improved self-reported strength and disability to a greater degree than the task-oriented approach alone [16]. However, a pilot trial conducted by Hung et al. [17] in the same year as a randomized clinical trial of the same group [18] showed different results with the interventions being essentially the same. The trial pointed to an increased effectiveness of bilateral robotic-assisted therapy mixed with traditional bilateral-assisted therapy in improving upper extremity mobility [18]. However, in later experiments, they found no difference in motor performance or functional recovery [17]. Similarly, Wu and colleagues reported that although BRT showed advantages in some kinematic outcome measures, these advantages did not translate into gains in daily function [19].

In conclusion, it remains questionable if BRT using a Bi-Manu-Track with an end-effector can assert clinically meaningful effects that are superior to conventional training or URT. Moreover, most BRT programs with good improvement in upper extremity function in stroke patients applied intensity of 90–105 min/time per day for 4–6 weeks. This high amount of time needed with the robot may result in a significant burden to patients and constrain the number of patients who can be trained on a given day.

For the above reasons, we conducted a three-arm randomized controlled trial investigating a different type of robot that used a three-dimensional end-effector, mainly acting on the shoulder and elbow as opposed to the forearm and wrist targeted by the Bi-Manu-Track. This robot is currently the first flexible steel rope drive, end-drive 3D upper limb rehabilitation robot in China [20], The robot can reduce shoulder abduction and trunk compensatory movements, while the weight loss support and range of motion calibration of the training task reasonably avoids sports injuries and pain, and helps patients perform better. In addition, with this new device, we also reduced the daily robot-assisted therapy time to about 60 min per day as opposed to the 90–105 min normally used with the Bi-Manu-Track. Based on this, we mainly included patients in the subacute stage, considering the relationship between the number of repeats and patient safety and neuroplasticity [21]. We aimed to determine whether bilateral or unilateral upper limb robot-assisted rehabilitation training using the new robotic device and reduced robotic therapy time was superior over regular therapy with regard to upper extremity motor function recovery, and neuromuscular improvement in post-acute hemiplegic stroke patients.

## 2. Methods

### 2.1. Design

This is a randomized, controlled, parallel, assessor-blinded, three-arm clinical trial with two intervention groups and one control group. The trial was prospectively registered at the Chinese Clinical Trials Registry (ChiCTR2100049484, http://www.chictr.org.cn/listbycreater.aspx, accessed on 2 August 2021). The study was conducted according to the tenets of the Declaration of Helsinki. This study was approved by the Ethics Committee of Southeast University Zhongda Hospital, Ethics No. (2021ZDSYLL091-P01; accessed on 8 March 2021).

### 2.2. Setting, Recruitment, and Consent

Subacute stroke patients who were hospitalized in the Rehabilitation Medicine Department of Southeast University Zhongda Hospital and met diagnostic criteria as defined by the Chinese Stroke Association Stroke Council Guideline Writing Committee [22] were enrolled between March 2021 and November 2022. In total, 161 patients were assessed for eligibility of which about 70 were deemed potentially eligible after prescreening of hospital records. All patients gave informed consent to this study and signed an informed consent form.

### 2.3. Participants

Inclusion criteria were: (1) aged 18–80 years old; (2) clear consciousness, no serious cognitive impairment, can follow the instructions to complete the corresponding assessment, Mini-Mental State Examination (MMSE) ≥20 points; (3) first onset, cerebral hemorrhage or cerebral infarction duration ≥2 weeks and ≤6 months; (4) stable clinical condition and co-morbid chronic diseases such as coronary heart disease, hypertension, diabetes, and hyperlipidemia well managed; (5) modified Ashworth spasticity assessment of the upper limb elbow joint major muscle tone ≤2 [23]; and (6) upper limb Brunnstrom of stage II, III, or IV. The exclusion criteria were: (1) cognitive or speech impairment affecting communication; (2) new infarcts or bleeding; (3) impaired movement of shoulder, elbow, or wrist due to trauma, soft tissue injury, fracture, frozen shoulder, joint contracture; (4) serious comorbidities such as digestive and endocrine system, or psychiatric disorders.

### 2.4. Interventions

The equipment used in this experiment was an upper limb rehabilitation robot provided by Nanjing ESTUN Company. The robot is a three-dimensional terminal robot that can perform shoulder joint flexion and abduction, elbow joint flexion, and extension. The two robot-assisted groups received 60 min per day, 6 days/week, for a total of 3 weeks. The patient is admitted to the hospital, the assessor evaluates the patient at baseline, and after the intervention, the assessor evaluates again. Participants in the URT and BRT groups received 30 min of robot-assisted training, followed by 30 min of functional tasks including reaching to move a cup, grasping and releasing blocks, picking up coins, barrel rolling training, wiping a table with two hands, and pegging board. Daily living training includes dressing, grooming, drinking, eating, etc. All groups received physical therapy. Details on intervention share are provided in Supplementary Table S2.

#### 2.4.1. Unilateral

URT used three modes of passive movement, assisted movement, and active movement. The therapist selected the appropriate treatment mode according to the patient’s functional condition. Each patient performed a total of three games, each for 10 min, which were aircraft wars, shooting mosquitoes, and ocean exploration (Supplementary Figure S1A).

#### 2.4.2. Bilateral

BRT is supposed to promote upper limb movement by driving the affected limb through the non-affected limb. Movement modes were the same as for unilateral robot-assisted training with the therapist selecting the mode according to the patient’s baseline function. Patients mainly performed rowing game training, which consisted of three parts: drumming, flagging, and oiling, 10 min, respectively (Supplementary Figure S1B).

#### 2.4.3. Conventional

Conventional rehabilitation treatment including functional electrical stimulation, Bobath technique, comprehensive training of hemiplegic limbs, maintenance training of a full range of joint motion, functional task training, self-care ability training in daily life (turning, sitting up, dressing, eating, etc.), combined with conventional drug treatment for underlying diseases. Patients received these therapies for 60 min per day, 6 days/week, for a total of 3 weeks.

### 2.5. Outcomes

#### 2.5.1. Primary Outcome

The primary outcome measure was the Fugl-Meyer Assessment–Upper Extremities Scale (FMA–UE). FMA–UE assesses upper limb motor function with thirty-three subitems. The evaluation 0 = completely immobile; 1 = can only complete part of the activity; or 2 = can perform the activity normally. The total score is 66 points and higher scores indicate better motor function [24].

#### 2.5.2. Secondary Outcomes

ADL was assessed with the Modified Barthel Index (MBI). The MBI consists of 10 items: control of urine and defecation, mobility, toileting, eating, bed and chair transfer, bathing, walking on flat ground, dressing, and walking up and down stairs. The total score is 100 points and higher scores indicate better motor function [25].

Surface electromyography (sEMG) was used to assess the biceps brachii bundle, triceps brachii bundle, anterior deltoid bundle, and middle deltoid bundle recruitment of motor units during muscle contraction. All four muscles were subjected to isometric contractions at maximal strength for 5 s three times, with 10 s rest between every test. Root mean square (RMS) and integrated electromyographic (iEMG) values were recorded and analyzed to judge the degrees of recruitment of motor units. The sEMG measurements were followed according to the recommendations of the SENIAM project (Surface EMG for Non-Invasive Assessment of Muscles) [26,27]. Motor evoked potential (MEP) was measured using a transcranial magnetic stimulation device (Magnetic Stimulation Therapy System, Youde Medical Equipment Co., Kaifeng, China), and the MEP protocol of measurements followed according to the practice guidelines of Groppa et al. [28]. The center of the “8” coil was placed on the area above the primary motor cortex with the handle positioned at 45° off the sagittal plane. MEPs were sampled until the location with the largest MEP was determined, using 100% intensity for 3 consecutive stimulations. If muscle compound action potential could be induced, latency and amplitude were recorded. For further analysis in this trial, MEP elicitation was coded binary (MEP induced vs. not).

### 2.6. Randomization

Permutated allocation sequences for 1:1:1 block randomization (block size 12–15) were computer-generated by an independent statistician. Allocation was concealed by central randomization and only revealed after baseline assessment through the call study center.

### 2.7. Blinding

The baseline visited for each potential study participant involved an assessor (occupational therapist) and an independent allocator (therapist). The assessor left the study site after the baseline measurement. The allocator then contacted the study center in the presence of the patient to disclose the allocation. Patients and therapists were asked not to disclose the allocation to assessors at any time during the study. For data analysts, the grouping of subjects was not known at the time of data analysis to ensure that analysts were blinded and that groupings were only disclosed after the data analysis was completed.

### 2.8. Sample Size Calculation

To detect a minimal clinical important difference (MCID) of 9 points in FMA–UE [29] with an sd of 10 [10,30], according to previous reports, 19 subjects per arm were needed to detect a statistically significant signal with an F-test of power 80% and alpha error of 5%. Assuming a dropout rate of 15%, the recruitment target was 22 subjects per arm.

### 2.9. Statistical Analysis

R 4.2.1 (R Foundation for Statistical Computing, Vienna, Austria) was applied for statistical analysis. To compare baseline differences among the 3 groups, the chi-square test was used for categorical data and analysis of variance (ANOVA) for continuous variables. The primary analysis was performed based on ITT using two-sided testing with a statistical significance level of α = 0.05. One-way analysis of covariance (ANCOVA) was used for the primary outcome FMA–UE [31]. Outcomes are reported as estimates of the least-squares mean (LSMEAN) with a 95% confidence interval (CI) for post-intervention change from baseline, and intervention effects are reported as least squares mean estimates with 95% CI for relative change values. Post-hoc comparisons were based on t-tests using Bonferroni correction for 3 tests, i.e., *p*-values < 0.0167 were considered statistically significant. The Huber–White sandwich estimator was used to obtain robust standard errors. All secondary outcomes were analyzed in the same fashion except for the MEP elicitation, which was coded binary (induced vs. not). The MEP elicitation rate was thus analyzed using a generalized linear model of the binomial family with a logit-link, and odds ratios (ORs) with 95% CIs are reported to indicate treatment effects in this case.

Sensitivity analyses consisted of per-protocol analysis (PP), and analysis after multiple imputations of missing data with chained equations (20 sets) [32,33]. In the latter model missing value imputation was performed while incorporating observations of auxiliary variables not included in the above model including sex, age, stroke type, stroke side, and stroke duration. Details on patterns of missing values are provided in Supplementary Table S3.

## 3. Results

**Figure 1:**
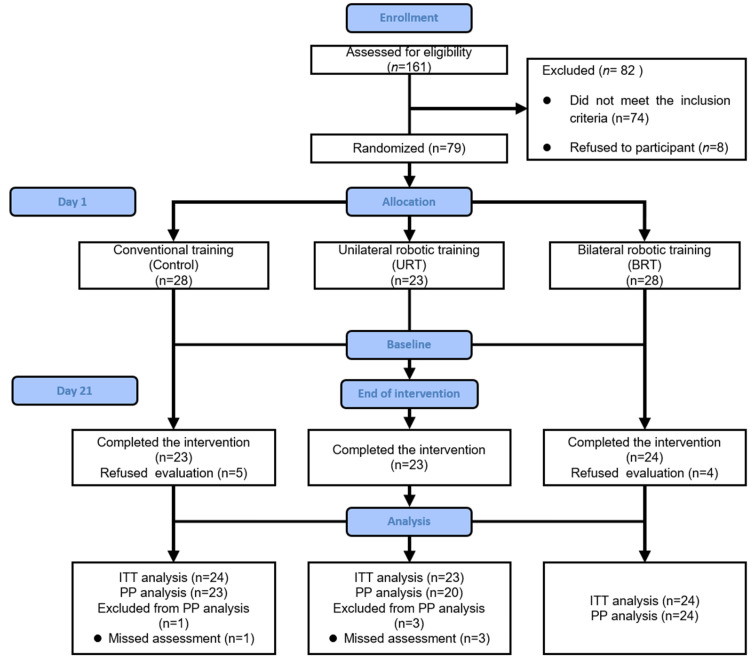
Consolidated Standards of Reporting Trials (CONSORT) flow diagram.

A CONSORT flowchart of the process used to identify and assess patients for inclusion is presented in Figure 1. Following a pre-screening of hospital records, 161 patients were contacted between March 2021 and November 2022 to further assess eligibility. Eighty-two of them were ineligible or refused consent. Seventy-nine patients who were randomized (four in the bilateral group and five in the control group) did not receive the assigned intervention as they decided to voluntarily withdraw before the start of the program. The absence of measurements for MEP in two patients and sEMG in two patients was due to temporary equipment failure. Eventually, 70 patients participated in the program with 23 in URT, 24 in BRT, and 23 in the control group. For more information on missing data within the exercise regimen, see online Supplementary Table S3.

Table 1 reports baseline characteristics of the study participants by intervention group. The mean age was 58.2 (SD 9.1), and the majority was male (n = 61, 87.1%). A total of 56 (80%) participants had an ischemic stroke, and 30 (42.9%) cases had a right-sided stroke site. Duration of stoke was 5.1 (SD 4.5) weeks on average. There was no significant difference in baseline demographic characteristics across the three treatment groups.

**Figure 2:**
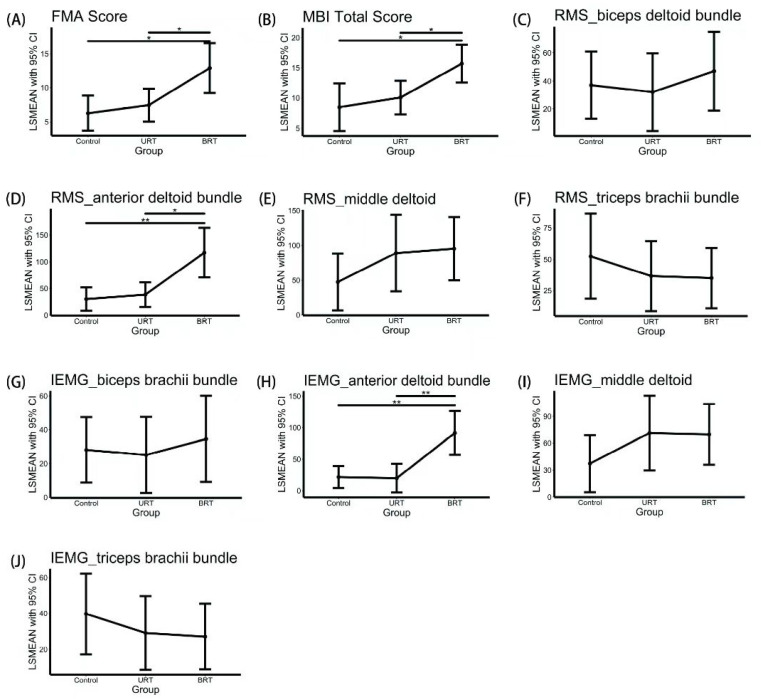
Post-hoc analysis of group comparisons of outcome variables using *t*-test adjusted with the Bonferroni method, *p*-values of post hoc tests were shown in Supplementary Table S3; * *p* < 0.0167, ** *p* < 0.0033. Post-hoc comparisons: (**A**) Fugl-Meyer Assessment–Upper Extremities (FMA-UE) score; (**B**) Mod-ified Barthel Index (MBI) score; (**C**) root mean square (RMS) of biceps brachii bundle; (**D**) RMS of anterior deltoid bundle; (**E**) RMS of middle deltoid; (**F**) RMS of triceps brachii bundle; (**G**) integrated electromyographic (iEMG) of biceps brachii bundle; (**H**) iEMG of anterior deltoid bundle; (**I**) iEMG of middle deltoid; (**J**) iEMG of triceps brachii bundle.

Table 2 gives an overview of pre- and post-intervention values, crude change, and adjusted treatment effects for all outcomes. Figure 2 depicts post hoc comparisons between-group by LSMEAN with 95% CI. Supplementary Table S4 gives *p*-values of post hoc tests based on t-tests using Bonferroni correction.

### 3.1. Primary Outcome Measure

FMA scores improved to varying degrees in all three groups after the intervention. LSMEAN and 95% CI of FMA scores for each group were 24.79 (22.23–27.35) in the control group, 25.97 (23.57–28.36) in the URT group, and 31.40 (27.74–35.07) in the BRT group, with statistically significant differences between groups in ANCOVA comparison (F = 4.328, *p* = 0.017); post hoc tests showed that the treatment effect was significantly higher in the BRT group than in the other two groups (BRT: 12.88, 95% CI 9.21–16.54; URT: 7.44, 95% CI 5.04–9.83; CT: 6.26, 95% CI 3.70–8.82. *p* = 0.0043 to control group, = 0.0159 to URT group), while URT was not statistically different from control (*p* = 0.504) (Figure 2 and Supplementary Table S4).

### 3.2. Secondary Outcome Measures

MBI differed significantly across groups (F = 5.266, *p* = 0.008) with BRT showing larger treatment effects with regard to ADL performance than the other two groups in post hoc tests (BRT: 15.68, 95% CI 12.55–18.80; URT: 10.07, 95% CI 7.31–12.84; control: 8.48, 95% CI 4.56–12.41. *p* = 0.0055 to control group, = 0.0091 to URT group). According to ANCOVA comparing RMS and iEMG of the biceps, anterior deltoid bundle, middle bundle, and triceps, after the intervention in the three groups, only RMS and iEMG of the anterior deltoid bundle differed statistically significantly between groups. Post-hoc tests showed that with 117.07 RMS, (95% CI 70.73–163.40; *p* = 0.0011 to control group, = 0.0034 to URT group) 91.61 iEMG (95% CI 56.68 −126.53; *p* = 0.0006 to control group, = 0.001 to URT group) changes in RMS and iEMG of the anterior deltoid bundle were statistically greater in the BRT group than in URT and controls. There were no statistically significant differences between URT and control. Using the control group as a reference, adjusted ORs for MEP elicitation were 1.82 for URT (95% CI 0.29–15.25) and 5.00 for BRT (95% CI 0.93–39.81). No statistically significant difference with control was detected though BRT was close to the threshold set for statistical significance (*p* = 0.54 for URT, *p* = 0.08 for BRT).

### 3.3. Sensitivity Analysis

The results of the sensitivity analyses are shown in Table 3. Treatment effects for the primary outcome differed little between the primary analysis based on ITT and PP analysis. Sensitivity analysis after multiple imputations for secondary outcomes also showed stable results. The primary analysis based on ITT appears to be robust.

## 4. Discussion

In this study, we compared the degree of improvement in upper limb function and ADL after stroke with two different robotic training modes, unilateral and bilateral. We used a new robotic device with a three-dimensional end-effector targeting the elbow and shoulder with a total therapy time of 60 min per day over a 3-week period. We found that BRT improved upper limb motor ability and performance in ADL to a greater extent than unilateral upper limb robotic training and conventional care control. Moreover, the recruitment of motor units in the anterior deltoid bundle was increased under bilateral training. Unilateral training did not differ statistically from control for any outcome.

As regards the primary outcome index of this study, statistical analysis showed that improvement in the FMA–UE score with BRT was statistically superior over unilateral robotic therapy and conventional therapy. In addition, On the Fugl-Meyer Assessment Scale in the robotic group, these differences reached minimal clinically meaningful values. In fact, BRT was the only training mode with which a clinically meaningful improvement in FMA–UE was achieved (9 points) [29] in our study. These results are in line with previous studies on bilateral robotic training that used the Bi-Manu-Track with an end-effector for about 90–105 min per day in addition to conventional therapy over a period of 4–6 weeks [14,15,16,17,34,35,36,37], while such effects had not been reported for the Bi-Manu-Track when a lower training intensity was applied [19].

Unlike other trials on upper limb robotic training in stroke populations that all used the Bi-Manu-Track end-effector robots, this study, for the first time, investigated a three-dimensional upper extremity end robot that targeted shoulder and elbow as opposed to forearm and wrist. This device elicits movement patterns of shoulder flexion and abduction as well as elbow flexion and extension while reducing the stress on the therapist through a three-dimensional movement trajectory. Our robot can reduce shoulder abduction and trunk compensatory movements [35] -, while the weight loss support and range of motion calibration of the training task reasonably avoids potential musculoskeletal injuries and helps patients perform better. In addition, previous studies provided higher-intensity training than that used in the present work, with the former using 90–105 min of training, five times per week. In addition, previous studies mostly involved patients in the later stages of stroke 1 to 2 years of onset and provided a higher intensity of training (90–105 min/session, 5 sessions/week) than the current work. Our study focused on subacute patients over a course of about one month, taking into account the relationship between the number of repetitions and patient safety and neuroplasticity [21]; in addition, according to patient feedback, more than 60 min would make them lose interest, cause fatigue, and delay other treatment time. In the previous phase, we adjusted the training time for patients by limiting the total length of the intervention to 60 min, Our finding that BRT improved upper limb function and ADL to a greater degree than URT or control may be related to the activation of brain mirror neurons through bilateral robotic therapy. In a large number of studies that used fMRI for evaluation, it has also been found that bilateral robot-assisted forearm training systems can increase interhemispheric connections (sensorimotor area) and intra-hemispheric connections (ipsilateral auxiliary motor area to the M1 area) [38]. Such increases may activate the ipsilesional primary motor cortex and supplementary motor area, thus rebalancing the abnormal interhemispheric transcallosal inhibition caused by stroke. Hence, the greater improvement in the pre-to-post difference in M1-M1 functional connectivity mediates the change and in this fashion can promote functional recovery of the upper limb.

Our study also suggests that in stroke patients with hemiplegia, unilateral upper limb robotic therapy have no obvious advantage compared with conventional treatment, though a number of previous studies and systematic reviews had reported that unilateral robotic therapy could be an alternative to conventional therapy. For patients diagnosed with stroke within six months, the effect of unilateral robot-assisted therapy in improving function and activities of daily living is similar to that of conventional therapy [2,13,39] However, Dehem et al., designed a unilateral robot-assisted treatment in combination with conventional therapy and found that the treatment was significantly more effective than conventional therapy alone [5]. Such inconsistencies may be related to the combination of robotic training with other varying treatment modalities, patient population, or type of robot. Based on the contrasting findings between our study and some of the above research views, we believe that URT combined with traditional therapy cannot be recommended for clinical application for the time being, at least in terms of treatment mode and dosage as these need to be further explored.

According to the “central-peripheral-central” closed-loop rehabilitation theory [40], surface EMG can observe the activation degree and contractile characteristics of peripheral muscles after stroke [41], and MEP can be used to assess the conductivity of central corticospinal tract [42,43,44], so as to explore the recovery mechanism of upper limb function after stroke. With the use of the sEMG-related indicators in this study, we found that RMS and iEMG of the anterior deltoid bundle of the BRT were significantly different from comparators after treatment. BRT’s increased effectiveness in improving the function of the proximal limb may be related to the movement trajectory of the robot being focused on forward flexion and abduction of the shoulder joint (Supplementary Figure S2). A pilot study shows that bilateral robot-assisted forearm training could be an optimal approach to improving proximal muscle power [37], which may be related to the proximal muscles driving the training modality of the robot used in this study. The robot used was a kind of terminal robot. Because the robot arm was moved mainly by the proximal muscles of the limb, the distal muscles were fixed during this training modality, so the proximal muscles activated more and earlier than the distal ones as detected by sEMG (Supplementary Figure S1 and Table S2).

Regarding the comparison of MEP, we found no significant differences among the three groups. The reason may be that the intervention measures in the short and medium term were not enough to change the state of MEP induction due to the limitation of the hospital stay in this experiment. In addition, this study found that it was difficult to induce MEP for moderate to severe upper limb movement disorders, and only in 21/70 patients could MEP be induced in the initial assessment. A lesser degree of recovery in patients is undetectable (Supplementary Figure S3). Longer-term intervention and follow-up may be needed to observe the predictive value of MEP testing. Similarly, Miller et al. reported that MEP did not have statistically significant changes in corticospinal excitability and transcallosal inhibition after a combined robotic and transcranial magnetic intervention [45]. These results suggest that BRT may promote the recruitment of motor neurons and improve the motor function of muscles, but not the conduction function of the corticospinal tract. The BRT can activate homologous muscle groups on the left and right sides by driving the affected side by the non-affected side, thus promoting the functional recovery of the upper limbs [46].

A significant improvement of upper limb function in patients with severe upper limb dysfunction (initial FMA < 30) after BRT training may suggest that patients with severe upper limb dysfunction may benefit more from BRT than those with mild to moderate dysfunction. Ranzani et al. performed a randomized controlled trial involving mainly mildly or moderately impaired (FMA–UE score > 30) patients with subacute stroke and found that motor recovery in the robot-assisted group was not inferior to that in the conventional care, suggesting that patients with minor deficits might have a ceiling effect on motor recovery [47]; thus, the effect of upper limb training was masked.

### Limitations

This trial has several limitations. First, the generalizability of the study is limited to stroke patients with cerebral hemorrhage or cerebral infarction in the post-acute phase ranging from ≥ 2 weeks to ≤ 6 months and cannot be extended to patients with ischemic stroke or those in the acute or chronic phase. Second, the neuroplasticity mechanism underlying the effects of unilateral and bilateral robots was not explored with functional imaging. At present, study results on URT show that the interhemispheric motor cortex connection at rest may be a potential marker of stroke recovery after rehabilitation [48]. However, connection patterns after URT may be different from that of BRT. Future research needs to explore the effect of unilateral vs. bilateral upper limb robotic training on the functional connectivity of related neuronal networks. Third, the two modes of robotic therapy were both combined with conventional upper limb training so that no conclusions can be drawn as to whether robotic training can replace conventional therapy. Fourth, this was a single-center trial and implementation of the intervention may vary by center, hence, the general applicability of BRT needs to be further confirmed through multi-center trials. Fifth, we lack the dosage comparison (30 min × 60 min), and the combination of robotic training + usual care. sixth, evidence shows [49] that, from a cost-benefit perspective, robot-assisted therapy seems to be an affordable and sustainable treatment measure. However, in China, for stroke patients, robotic therapy is not covered by medical insurance and may thus induce an additional economic burden on patients and families. Studies on the cost-effectiveness of robot-assisted therapy are thus needed in China to bring about insurance policy changes to cover treatment costs.

## 5. Conclusions

Our results indicate that adding BRT using a three-dimensional end-effector type robot targeting shoulder and elbow to conventional therapy can improve upper limb motor ability and performance in ADL in a clinically meaningful way, while the provision of URT in addition to conventional therapy does not appear to be clinically or statistically superior over of conventional therapy only. Therapy time can be reduced to 60 min per day with this new type of BRT. Electrophysiological results suggest that the BRT of the upper limb robot increases the recruitment of motor neurons rather than improving the conduction function of the corticospinal tract.

## Figures and Tables

**Table 1:** Demographic and clinical characteristics of treated subjects.

Baseline Characteristics	Total, N = 70	Control, N = 23	URT, N = 23	BRT, N = 24	*p* Value *
Sex, N (%)					1
Male	61 (87.1%)	20 (87%)	20(87%)	21 (87.5%)	
Female	9 (12.9%)	3 (13%)	3 (13%)	3 (12.5%)	
Age, Mean (SD)	58.2(9.1)	58.9(10.3)	56.7 (8.9)	59.0 (8.3)	0.6
Stroke type, N (%)					0.9
Ischemic	56 (80%)	18(78.3%)	19 (82.6%)	19 (79.2%)	
Hemorrhage	14 (20%)	5 (21.7%)	4 (17.4%)	5(20.8%)	
Stroke side, N (%)					0.8
left	40 (57.1%)	12 (52.2%)	14 (60.9%)	14 (58.3%)	
right	30 (42.9%)	11 (47.8%)	9(39.1%)	10 (41.7%)	
Stroke weeks, Mean (SD)	5.1 (4.5)	5.0 (3.6)	4.7(4.3)	5.5(5.5)	0.8

BRT, bilateral robotic training; SD, standard deviation; URT, unilateral robotic training. * chi-square test for categorical data and ANOVA for continuous variables.

**Table 2:** Clinical outcome measures and inferential statistics.

Measurements	Baseline	Post Intervention	Between-Group Comparison				Treatment Effect
	Mean ± SD */ N (%) ^§^	Mean ± SD */N (%) ^§^	Adjusted Mean ^†^/OR ^‡^	95% CI	F Value (df)	*p* Value	Adjusted Mean with 95% CI
**Primary Outcome**							
FMA–UE							
Control	18.57 ± 11.47	24.83 ± 15.10	24.79	22.23–27.35	4.328 (66)	0.017	6.26 (3.70–8.82)
URT	17.91 ± 11.56	25.30 ± 13.99	25.97	23.57–28.36			7.44 (5.04–9.83)
BRT	19.08 ± 9.17	32.00 ± 11.44	31.40	27.74–35.07			12.88 (9.21–16.54)
**Secondary Outcome**							
MBI Total Score							
Control	54.74 ± 13.44	63.13 ± 16.31	62.75	59.42–66.09	5.266 (66)	0.008	8.48 (4.56–12.41)
URT	53.52 ± 12.82	63.74 ± 11.54	64.34	61.01–67.68			10.07 (7.31–12.84)
BRT	54.54 ± 11.67	70.17 ± 10.27	69.95	66.69–73.21			15.68 (12.55–18.80)
RMS_biceps brachii bundle							
Control	120.76 ± 81.50	157.86 ± 110.82	162.55	138.60–186.50	0.283 (64)	0.755	36.99 (13.04–60.94)
URT	128.30 ± 96.44	160.25 ± 95.57	157.57	129.82–185.33			32.01 (4.26–59.77)
BRT	127.76 ± 76.76	174.72 ± 104.55	172.57	144.35–200.79			47.01 (18.79–75.23)
RMS_anterior deltoid bundle							
Control	148.47 ± 119.00	177.61 ± 131.79	170.77	148.97–192.58	5.664 (64)	0.006	30.05 (8.25–51.86)
URT	139.19 ± 114.10	177.70 ± 115.11	179.05	156.03–202.07			38.33 (15.31–61.35)
BRT	134.63 ± 75.72	252.41 ± 116.69	257.79	211.45–304.12			117.07 (70.73–163.40)
RMS_middle deltoid							
Control	159.09 ± 103.13	206.04 ± 156.59	202.11	156.84–247.38	1.317 (64)	0.275	47.42 (6.37–88.47)
URT	134.80 ± 108.52	225.89 ± 163.69	243.63	196.02–291.25			88.94 (33.81–144.07)
BRT	167.88 ± 113.33	261.69 ± 112.02	249.92	205.50–294.35			95.23 (49.71- 140.75)
RMS_triceps brachii bundle							
Control	73.40 ± 54.73	126.23 ± 100.02	135.55	107.21–163.88	0.458 (64)	0.635	52.42 (18.58–86.26)
URT	77.41 ± 63.15	114.25 ± 90.48	119.72	90.13–149.30			36.59 (8.74–64.45)
BRT	97.44 ± 89.06	131.85 ± 95.84	118.14	90.29–146.00			35.02 (10.98–59.06)
iEMG_biceps brachii bundle							
Control	93.87 ± 64.75	122.05 ± 87.77	125.06	105.61–144.51	0.148 (64)	0.863	28.13 (8.68–47.58)
URT	100.15 ± 74.21	125.24 ± 74.96	122.08	99.50–144.67			25.15 (2.57–47.73)
BRT	97.06 ± 61.55	131.62 ± 90.08	131.49	105.96–157.02			34.56 (9.02–60.09)
iEMG_anterior deltoid bundle							
Control	118.28 ± 95.42	138.37 ± 101.79	132.09	114.51–149.68	6.812 (64)	0.002	21.69 (4.10–39.27)
URT	109.22 ± 90.30	129.43 ± 67.98	130.38	107.50–153.26			19.97 (−2.91–42.86)
BRT	103.90 ± 56.95	196.83 ± 96.77	202.01	167.09–236.94			91.61 (56.68–126.53)
iEMG_middle deltoid							
Control	127.10 ± 88.57	161.80 ± 125.66	157.49	117.63–197.35	1.865 (64)	0.163	36.79 (−2.98–76.56)
URT	102.84 ± 78.95	156.02 ± 80.47	168.03	126.07–210.00			47.34 (22.54–72.13)
BRT	130.19 ± 89.27	214.99 ± 118.95	208.60	169.53–247.67			87.90 (40.29–135.52)
iEMG_triceps brachii bundle							
Control	52.24 ± 40.46	93.25 ± 76.31	97.71	70.05–125.37	0.104 (64)	0.901	38.06 (6.65–69.48)
URT	53.45 ± 44.97	85.87 ± 67.30	89.60	60.70–118.51			29.96 (3.72–56.19)
BRT	72.17 ± 64.3	97.79 ± 73.30	90.25	62.98–117.52			30.60 (7.39–53.82)
MEP (response)							
Control	8 (34.8%)	11 (47.8.0%)	Reference				
URT	7 (30.4%)	11 (47.8.0%)	1.82	0.29–15.25		0.54	
BRT	6 (25.0%)	15 (62.5%)	5	0.93–39.81		0.08	

* Continuous variables in the outcome indicators are expressed as Mean ± SD. ^§^ For MEP, the frequency and percentage of MEP elicitation are provided. ^†^ Continuous outcome variables were analyzed by covariance analysis, adjusted for baseline, and estimated least squares means (LSMEAN) and 95% CI for each group after intervention. ^‡^ MEP (elicitation: elicitation here means outcome = 1) outcomes were estimated using longitudinal logistic regression, linking logit functions to adjust the baseline and yielding OR values with 95% CI. BRT, bilateral robotic training; CI credit interval; FMA–UE, Fugl-Meyer Assessment–Upper Extremities; iEMG, integrated electromyographic; MBI, Modified Barthel Index; MEP, Motor Evoked Potential; RMS, root mean square; SD, standard deviation; URT, unilateral robotic training.

**Table 3:** Results of sensitivity analysis.

Measurement	ITT Based Primary Analysis (N_Control_ = 24, N_URT_ = 23, N_BRT_ = 24)		PP Based Analysis * (N_Control_ = 23, N_URT_ = 20, N_BRT_ = 24)		ITT, MI with Chained Equations ^§^ (N_Control_ = 24, N_URT_ = 23, N_BRT_ = 24)	
	Estimates of Treatment Effects (95%CI)/OR (95%CI)	*p* Value	Estimates of Treatment Effects (95%CI)/OR (95%CI)	*p* Value	Estimates of Treatment Effects (95%CI)/OR (95%CI)	*p* Value
**Primary Outcome**						
FMA–UE						
Control	6.26 (3.70–8.82)	0.017	6.53 (3.89–9.17)	0.022	-	-
URT	7.44 (5.04–9.83)		7.38 (4.68–10.09)		-	
BRT	12.88 (9.21–16.54)		12.90 (9.25–16.56)		-	
**Secondary Outcomes**						
MBI						
Control	8.48 (4.56–12.41)	0.008	8.97 (4.93–13.01)	0.014	-	-
URT	10.07 (7.31–12.84)		10.07 (7.01–13.12)		-	
BRT	15.68 (12.55–18.80)		15.68 (12.56–18.79)		-	
RMS_biceps brachii bundle						
Control	36.99 (13.04–60.94)	0.755	40.12 (16.34–63.91)	0.824	36.96 (12.96–60.96)	0.491
URT	32.01 (4.26–59.77)		34.01 (4.97–63.04)		29.60 (4.08–55.13)	
BRT	47.01 (18.79–75.23)		47.00 (18.75–75.26)		47.01 (18.80–75.22)	
RMS_anterior deltoid bundle						
Control	30.05 (8.25–51.86)	0.006	30.69 (7.92–53.46)	0.006	30.15 (8.36–51.94)	0.001
URT	38.33 (15.31–61.35)		39.60 (15.62–63.58)		41.53 (20.10–62.97)	
BRT	117.07 (70.73–163.40)		116.66 (70.45–162.87)		117.14 (70.82–163.45)	
RMS_middle deltoid						
Control	47.42 (6.37–88.47)	0.275	50.24 (7.76–92.72)	0.358	47.61 (6.68–88.55)	0.205
URT	88.94 (33.81–144.07)		86.04 (28.50–143.58)		90.55 (39.38–141.71)	
BRT	95.23 (49.71–140.75)		95.04 (49.58–140.50)		95.44 (49.99–140.90)	
RMS_triceps brachii bundle						
Control	52.42 (18.58–86.26)	0.635	54.89 (20.00–89.77)	0.505	52.31 (18.44–86.19)	0.493
URT	36.59 (8.74–64.45)		33.00 (4.71–61.29)		35.31 (10.14–60.48)	
BRT	35.02 (10.98–59.06)		34.91 (10.83–58.99)		34.94 (10.91–58.97)	
iEMG_biceps brachii bundle						
Control	28.13 (8.68–47.58)	0.863	30.54 (11.15–49.92)	0.915	28.10 (8.60–47.61)	0.545
URT	25.15 (2.57–47.73)		26.99 (3.39–50.60)		23.51 (2.83–44.20)	
BRT	34.56 (9.02–60.09)		34.56 (8.99–60.12)		34.55 (9.03–60.07)	
iEMG_anterior deltoid bundle						
Control	21.69 (4.10–39.27)	0.002	22.31 (3.92–40.70)	0.003	21.15 (4.07–38.23)	0.000
URT	19.97 (−2.91–42.86)		20.43 (−3.35–44.21)		23.43 (1.23–45.63)	
BRT	91.61 (56.68–126.53)		91.07 (56.10–126.03)		91.58 (56.69–126.48)	
iEMG_middle deltoid						
Control	36.79 (−2.98–76.56)	0.163	39.40 (6.43–72.36)	0.398	37.10 (5.37–68.84)	0.306
URT	47.34 (22.54–72.13)		68.77 (25.29–112.25)		73.46 (35.29–111.63)	
BRT	87.90 (40.29–135.52)		69.60 (35.64–103.56)		69.65 (35.61–103.69)	
iEMG_triceps brachii bundle						
Control	38.06 (6.65–69.48)	0.901	41.60 (18.52–64.69)	0.499	39.62 (17.32–61.92)	0.636
URT	29.96 (3.72–56.19)		26.16 (5.56–46.76)		28.90 (9.89–47.92)	
BRT	30.60 (7.39–53.82)		27.13 (8.79–45.46)		27.00 (8.83–45.17)	
MEP (response)						
Control	Reference		Reference		Reference	
URT	0.29–15.25	0.54	1.10 (0.17–7.20)	0.9	1.82 (0.36–10.62)	0.48
BRT	0.93–39.81	0.08	3.67 (0.81–20.50)	0.1	4.00 (0.90–22.21)	0.08

* Per-protocol-based analysis included only patients who completed the study according to the intervention protocol specified at randomization entry; patients who did not receive or complete the study process according to the established protocol were excluded from the analysis. ^§^ Based on multiple interpolations using chained equations, assuming missing data at random. The interpolation model included all outcomes and the following predictor variables: sex, age, type of stroke, side of stroke, and length of stroke (week). BRT, bilateral robotic training; CI credit interval; FMA–UE, Fugl-Meyer Assessment-Upper Extremities; iEMG, integrated electromyographic; ITT, intention-to-treat; MBI, Modified Barthel Index; MEP, Motor Evoked Potential; MI, multiple imputations; PP, per protocol; RMS, root mean square; SD, standard deviation; URT, unilateral robotic training.

## Data Availability

The raw data supporting the conclusions of this article will be made available by the authors, without undue reservation.

## Supplementary Materials

The following supporting information can be downloaded at: https://www.mdpi.com/article/10.3390/jcm12082950/s1, Figure S1: (A): Unilateral upper limb robot-assisted rehabilitation; (B): Bilateral upper limb robot-assisted rehabilitation; Figure S2: (A,B): anterior deltoid bundle before and after treatment; Figure S3: (A,B): contralateral brain before and after treatment; (C,D): ipsilateral brain before and after treatment; Table S1: Detailed characteristics of previous bilateral robot-assisted training randomized controlled trials; Table S2: Specific description of intervention methods; Table S3: Missing data patterns for outcome variables (1 = complete, 0 = missing); Table S4: Post hoc test results.
