# Supplementary material for: Effects of Uni- vs. Bilateral Upper Limb Robot-Assisted Rehabilitation on Motor Function, Activities of Daily Living, and Electromyography in Hemiplegic Stroke: A Single-Blinded Three-Arm Randomized Controlled Trial
Source: J Clin Med. 2023 Apr 18;12(8):2950. doi: 10.3390/jcm12082950 (PMC10143606; doi:10.3390/jcm12082950)
Supplement: Supplementary file 1 [file jcm-12-02950-s001.zip › jcm-2209460-supplementary.pdf]

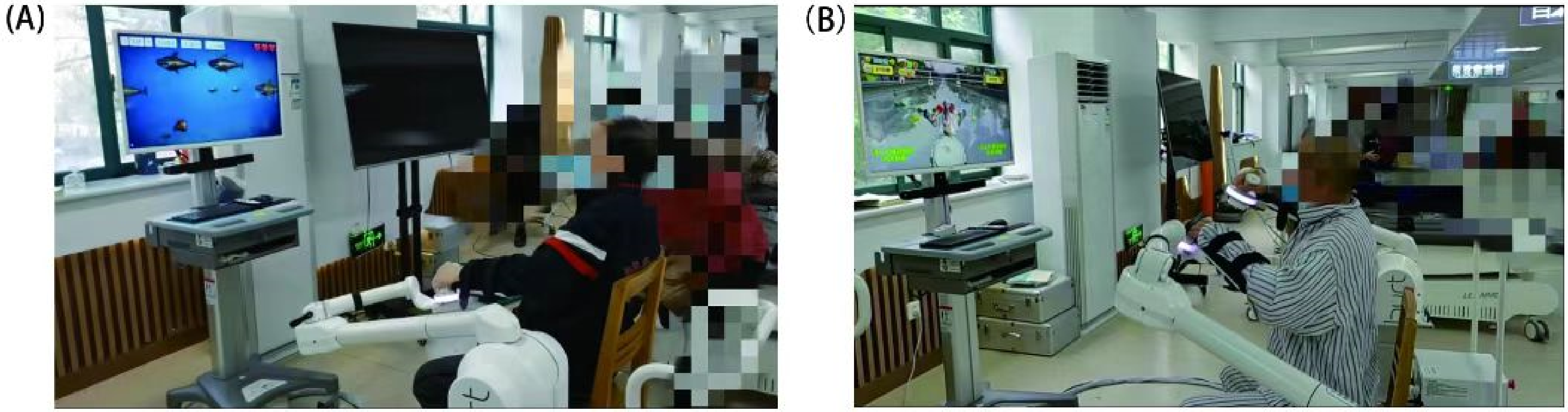

Supplementary Figure S1. | (A): Unilateral upper limb robot-assisted rehabilitation; (B): Bilateral upper limb robot-assisted rehabilitation.

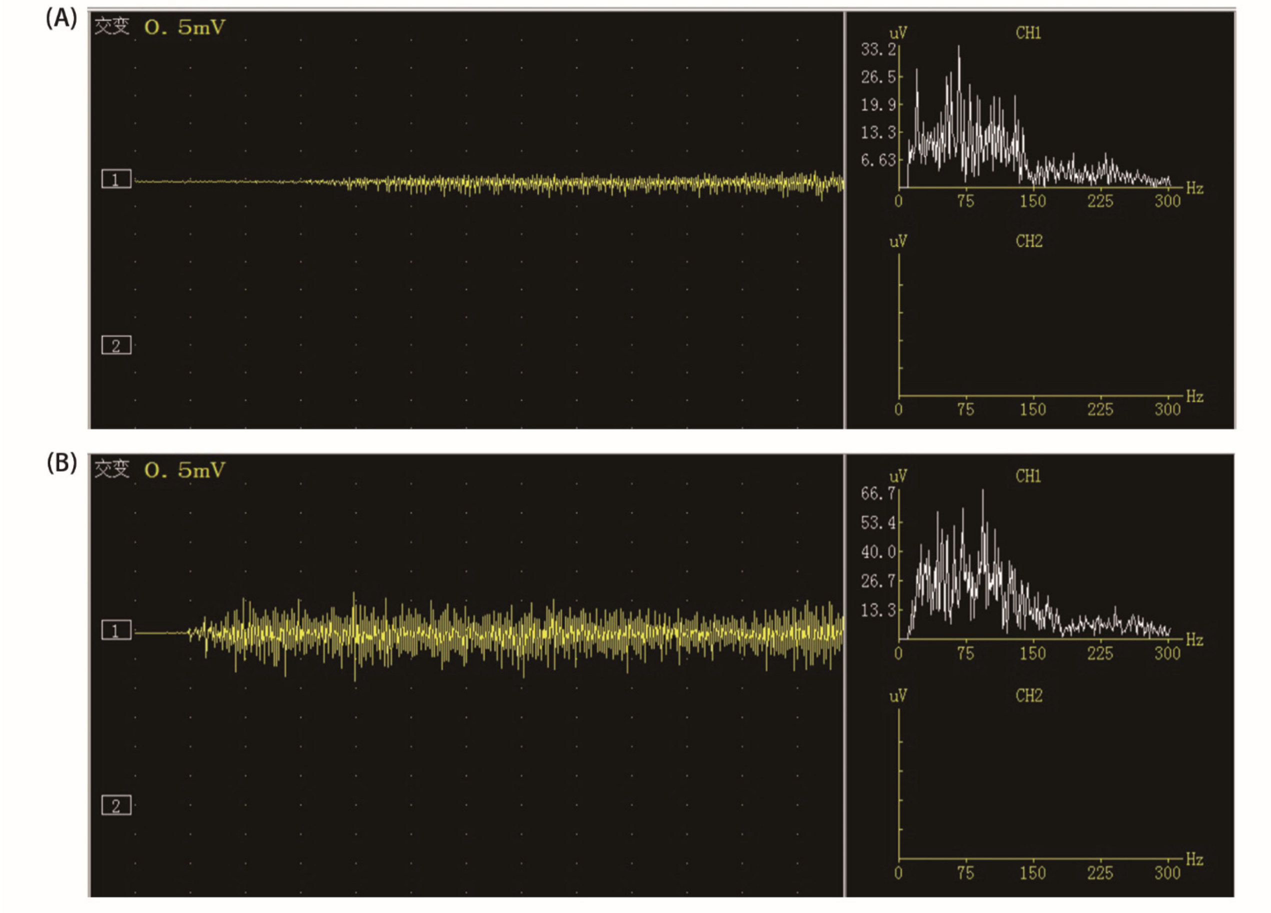

Supplementary Figure S2. | **(**A) and (B): anterior deltoid bundle before and after treatment.

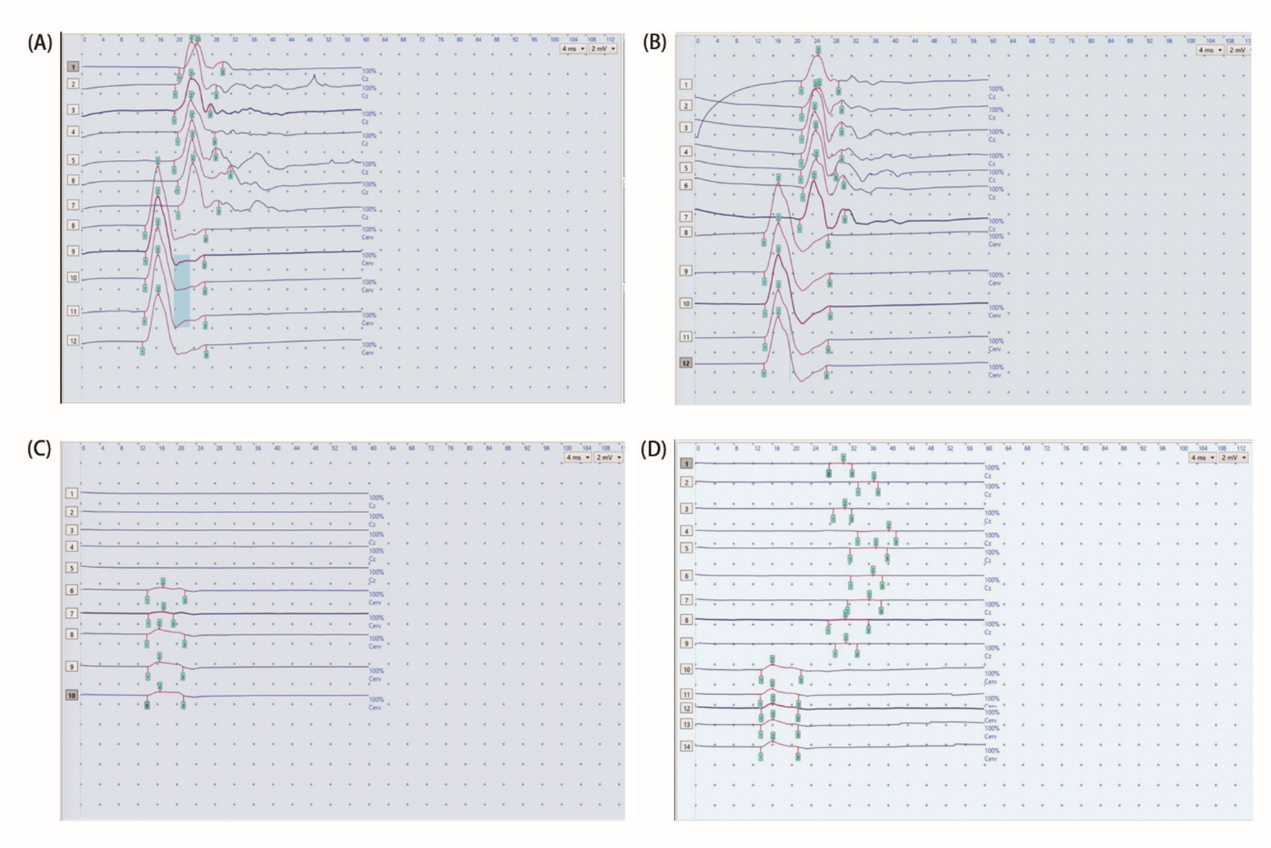

Supplementary Figure S3. | ((A) and (B): contralateral brain before and after treatment; (C) and (D): ipsilateral brain before and after treatment.

Supplementary Table S1. | Detailed characteristics of previous bilateral robot-assisted training randomized controlled trials.

Study	Population	Rehabilitation intervention	Robot type	Training mode	Training part of upper extremity	Intensity	Comparison group (s)	Outcomes and findings (Between-group comparison)	Authors' conclusions
**Wu et al. (2012)**	post-stroke mild-to-moderate motor impairment (initial score of 26 to 66 on the FMA for the upper limb)	BRT (n=14): about 30 min in modes 1 and 2 and about 10 min in mode 3 for each type of movement<br>They received 75 to 80 minutes of BRT, followed by 15 to 20 minutes of unilateral and bilateral functional training and 5 minutes of tone normalization at the end of therapy, if necessary.	Bi-Manu-Track<br><br>End-effector	mode 1 (passive–passive mode)<br>mode 2 (active–passive mode)<br>mode 3 (active–active mode)	forearm pronation–supination and wrist flexion–extension	90 to 105 min/day, 5 days/week, for 4 weeks	Bilateral assisted training, BAT (n=14): passive (helped by therapists) or active training. Among the tasks were lift 2 cups, stack 2 checkers, reach forward or upward to move blocks, grasp and release 2 towels, and manipulate 2 coins simultaneously by each hand. The TBAT group also practiced 15 to 20 minutes of functional training and 5 minutes of tone normalization at the end of therapy, if necessary.<br>CT (n=14): CT group involved weight bearing, stretching, strengthening of the	FMA: total score: -; Proximal: -; Distal: BAT superior than CT;<br>MAL: AOM: -; QOM: -;<br>SIS: total score, strength, physical function: BRT superior than CT; Memory, Emotion, Communication, ADL/IADL, Mobility, Hand function, Participation: -.	This is the first study to compare bilateral arm training mediated by a therapist versus a robot in improving motor control, functional performance, and quality of life in patients with stroke. These findings suggest that TBAT might uniquely improve temporal efficiency, smoothness, and trunk compensation of reaching movement and motor impairment of the distal part of the UL. Robot-assisted BAT may be a more compelling approach to improve shoulder flexion range of motion and quality of life related to paretic UL function.

							paretic arms, coordination, unilateral and bilateral fine motor tasks, balance, and compensatory practice on functional tasks		
**Wu et al. (2013)**	post-stroke mild-to-moderate motor impairment (initial score of 20 to 66 on the FMA for the upper limb)	BRT (n=18): BRT (75-80 min/session) + functional training (15-20 min/session)<br>BRT includes 75 to 85 minutes of robot-assisted arm training during which each patient practiced 300 to 400 repetitions of the elbow and wrist cycles in mode 1 and mode 2 and practiced 50 to 80 repetitions in mode 3	Bi-Manu-Track<br><br>End-effector	mode 1 (passive–passive mode)<br>mode 2 (active–passive mode)<br>mode 3 (active–active mode)	forearm pronation–supination and wrist flexion–extension	90 to 105 min/day, 5 days/week, for 4 weeks	URT (n=18): URT (75-80 min/session) + functional training (15-20 min/session)<br>URT: 3 modes were modified, during mode 1 training, the robotic device provided full assistance for the paretic arm; during mode 2 training, the paretic arm moved the handle independently; and during mode 3 training, the paretic arm moved the handle against a resistance determined by the therapist through the entire movement	WMFT: URT superior than BRT<br>WMFT-FAS, MAL, and ABILHAND scores: -	BRT and URT resulted in differential improvements in specific UE/trunk performance in patients with stroke. BRT elicited larger benefits than URT on reducing compensatory trunk movements at the beginning of reaching. In contrast, URT produced better improvements in UE temporal efficiency. These relative effects on movement kinematics, however, did not translate into differential benefits in daily functions.

							CT (n=17): CT (90-105 min/session)<br>CT: The therapeutic activities in the CT group included weight bearing, stretching, and strengthening of the paretic arm, coordination tasks, unilateral and bilateral fine motor tasks, and balance activities.		
**Hsieh et al. (2016)**	post-stroke mild-to-moderate motor impairment (initial score of 20 to 66 on the FMA for the upper limb)	BRT (n=17): BRT (75-90 min/session) + functional training (15-20 min/session)<br>5-10 min warm-up + 600 to 800 repetitions of the passive–passive and active–passive modes for 15 to 20 min and 150 to 200 repetitions of the active-active mode for 3 to 5 min + 15 to 20 minutes of functional training	Bi-Manu-Track<br><br>End-effector	mode 1 (passive–passive mode)<br>mode 2 (active–passive mode)<br>mode 3 (active–active mode)	forearm pronation–supination and wrist flexion–extension	90 to 105 min/day, 5 days/week, for 4 weeks	BRT + modified constraint-induced therapy (mCIT) (n=17): BRT (75-90 min/session) + functional training (15-20 min/session) + use a mitt to restrict the unaffected hand (6 h/day)<br>BRT program same as BRT group. mCIT: Using a mitt to restrict the unaffected hand for 6 h each day, use the other side to finish the functional	WMFT-FAS: RT+mCIT↑; WMFT-TIME: -; NEADL: RT+mCIT↑; FIM: -	BRT + mCIT could be an effective approach to improve stroke rehabilitation outcomes, achieving better motor control strategies, motor function, and functional independence of instrumental activities of daily living.

							tasks. (The shaping techniques involved individualized task selection, graded task difficulty, verbal feedback, prompting, physical assistance with movements, and modeling. The level of challenge was adapted according to the patient's ability and progress)		
**Hsieh et al. (2017)**	Post unilateral stroke mild-to-moderate motor impairment (initial score of 20 to 50 on the FMA for the upper limb)	(Priming, defined as a change in behavior on the basis of previous stimuli, is an emerging strategy to facilitate motor relearning in neurorehabilitation.) BRT (n=16): Bilateral robotic priming (40-45 min/session) + task-oriented training (40-45 min/session) Examples of functional tasks were (1) filling a bottle from a fountain,	Bi-Manu-Track<br><br>End-effector	mode 1 (passive–passive mode)<br>mode 2 (active–passive mode)<br>mode 3 (active–active mode)	forearm pronation–supination and wrist flexion–extension	90 min/day, 5 days/week, for 4 weeks, 20 sessions in total	CT (n=15): task-oriented training (40-45 min/phase, 2 phases/session)<br>phase one, three specific tasks were targeted and intensively practiced for 240 to 300 purposeful repetitions. The characteristics of the functional tasks in this phase were relatively simple and with fewer steps, allowing patients to	FMA-UE: -; BBT: -; Grip: -; mRS: -; FIM: -; Actigraphy: -;<br>SIS: Strength: BRT priming ↑; Hand function, ADL/IADL, Mobility: -; Fatigue: -	Bilateral priming combined with the task-oriented approach elicited more improvements in self-reported strength and disability degrees than the task-oriented approach by itself. Further large-scale research with at least 31 participants in each intervention group is suggested to confirm the study findings.

		transferring to the therapy room, and drinking water from the bottle; (2) wipe the table with a cloth, and (3) folding towels and putting them in the drawers. Each functional activity was practiced for 15 to 20 minutes.					perform mass repetitions of the tasks. The tasks involved reach to grasp, object manipulation, and pinch and grip movements, and included such tasks as sorting blocks or cards, putting pegs into holes, stacking cones, flipping cards, and stacking checkers.<br>The treatment principles and programs of phase two in the control group were the same as those in the experimental group.		
**Hung et al. (2019a)**	post-stroke mild-to-moderate motor impairment (initial score of 18 to 56 on the FMA	BHT (n=10): BRT (40-45 min/session) + BAT (40-45 min/session) + home-program teaching (10 min/session)<br>BRT training mode same as URT	Bi-Manu-Track<br><br>End-effector	mode 1 (passive–passive mode)<br>mode 2 (active–passive mode)	forearm pronation-supination andwrist flexion-extension patterns	90 min/whole session, 3 sessions/week, 6 weeks	UHT (n=9): URT (40-45 min/session) + mCIT (40-45 min/session) + home-program teaching (10 min/session)<br>URT intervention protocol provided forearm pronation–	FMA, FMA-proximal, FMA-distal, CAHAI: -;<br>GAS: BHT superior than UHT and CT	Hybrid therapies are safe and applicable interventions for chronic stroke and favorable for improving individual functional goals. Treatment effects on motor recovery and functional activity

	for the upper limb)	BAT contained two to three tasks that used both hands, such as lifting a big box, throwing a basketball, and folding clothes. Each task was repeated 30 to 50 times.	mode 3 (active–active mode)		supination and wrist flexion-extension movement patterns. Each pattern included passive movement practice (the affected UE passively moved by the device) for 350 to 400 repetitions and active movement practice for 200 to 350 repetitions by the participant's affected UE.<br>mCIT contained two to three tasks, such as using a spoon, throwing a baseball, and holding a glass. Each task was repeated 30 to 50 times. Participants were asked to constrain their unaffected hand by a mitt for a target of 5 hr daily at home<br><br>RT (n=11): RT (80-90 min/session) + home-program		might be similar among the three groups.

							teaching (10 min/session); both URT and BRT, the repetition for each pattern ranged from 450 to 500 times in both unilateral and bilateral mode.		
**Hung et al. (2019b)**	post-stroke mild-to-moderate motor impairment (initial score of 18 to 56 on the FMA for the upper limb)	BHT (n=15): BRT (40-45 min/session) + BAT (40-45 min/session) + home-program teaching (10 min/session)<br>BRT training mode same as URT<br>BAT contained two to three tasks that used both hands, such as lifting a big box, throwing a basketball, and folding clothes. Each task was repeated 30 to 50 times.	Bi-Manu-Track<br><br>End-effector	mode 1 (passive–passive mode)<br>mode 2 (active–passive mode)<br>mode 3 (active–active mode)	forearm pronation-supination andwrist flexion-extension patterns	90 min/whole session, 3 sessions/week, 6 weeks	UHT (n=14): URT (40-45 min/session) + UAT (40-45 min/session) + home-program teaching (10 min/session)<br>URT intervention protocol provided forearm pronation–supination and wrist flexion-extension movement patterns. Each pattern included passive movement practice (the affected UE passively moved by the device) for 350 to 400 repetitions and active movement practice for 200 to 350 repetitions by the participant's affected UE.	*Baseline to post-intervention:*<br>FMA: total: BHT superior than UHT; FMA-proximal: -; FMA- distal: BHT superior than UHT;<br>SIS: -;<br>WMFT-time, WMFT-FAS: -;<br>NEADL: Mobility: RT superior than BHT; Kitchen, Living affairs, Leisure: -;<br><br>*Baseline to follow-up:*<br>FMA: total: BHT superior than UHT; FMA-proximal: -; FMA- distal: BHT superior than UHT and RT;<br>SIS: -;	BHT was more effective for improving upper extremity motor function, particularly distal motor function at follow-up, and individuals in the RT group demonstrated improved functional ambulation post intervention.

							UAT contained two to three tasks, such as using a spoon, throwing a baseball, and holding a glass. Each task was repeated 30 to 50 times. Participants were asked to constrain their unaffected hand by a mitt for a target of 5 hr daily at home<br>RT (n=15): RT (80-90 min/session) + home-program teaching (10 min/session); both URT and BRT, the repetition for each pattern ranged from 450 to 500 times in both unilateral and bilateral mode.		
**Liao et al. (2012)**	post-stroke mild-to-moderate motor impairment (initial score of 20 to 56	BRT: BRT (75-90 min/session) + functional training (15 min)<br>BRT: 300 to 400 forearm cycles, totaling 600–800	Bi-Manu-Track<br><br>End-effector	mode 1 (passive–passive mode): both arms controlled by the robot	forearm pronation-supination andwrist flexion-extension patterns	90 to 105 min/day, 5 days/week, for 4 weeks	CT: occupational therapy (therapy time and does match with BRT) + functional training (15 min)<br>Occupational therapy techniques included	FMA: BRT↑; FIM: -;<br>MAL: AOU: BRT↑; QOM: BRT↑;<br>ABILHAND: BRT↑;<br>Accelerometer: BRT↑;	Symmetrical and bilateral robotic practice, combined with functional task training, can significantly improve motor function, arm activity, and self-

	on the FMA for the upper limb)	repetitions of mode 1 and mode 2, and 150–200 repetitions of mode 3, respectively, for the forearm and the wrist movements. If the affected arm was able to actively perform forearm pronation–supination or wrist flexion–extension, mode 2 was adjusted to mode 4 to encourage more active movements of the affected arm.<br>Functional training included twisting a towel, turning a key in the lock, opening a jar, carrying heavy objects, using chopsticks, writing, folding clothes, picking up coins, turning a door knob, and so on.	mode 2 (passive-active mode): with the non-affected arm driving the affected arm<br>mode 3 (active–active mode): with the affected arm actively moving against the initial resistance, then both arms cooperating to achieve movement<br>mode 4 (active–passive mode): with the affected arm actively executing		neurodevelopmental techniques, with emphasis on functional task training		perceived bilateral arm ability in patients late after stroke.

				the training cycles					
**Hsu et al. (2019)**	post-stroke	BRT: sensorimotor stimulation session - repetitive upper limb range of motion exercises (10 min/session) + BRT (40 min/session)<br>BRT: a minimum of 400 robot-facilitated repetitions of the wrist flexion/extension as well as 400 repetitions of forearm supination/pronation movement	Bi-Manu-Track<br><br>End-effector	mode 1 (passive–passive mode)<br>mode 2 (active–passive mode)<br>mode 3 (active–active mode)	forearm pronation-supination andwrist flexion-extension patterns	50 min/session, 3 sessions/week, for 4 weeks	usual care with consisted of 10-minute sensorimotor stimulation program and 40 minutes of therapist-facilitated task-specific training for the affected limb.	*Baseline to post-intervention:*<br>MAL: AOU: -; QOM: -;<br>FMA: Shoulder/Elbow/Forearm: -; Wrist: BRT↑; Hand: CT↑; Coordination: -; total: -;<br>RMS: SF (first 30% phase): -; SF (late 30% phase): -; WE (first 30% phase): BRT↑; WE (late 30% phase): -; WF (first 30% phase): -; WF (late 30% phase): -;<br>*Baseline to follow-up:*<br>MAL: AOU: BRT↑; QOM: -;<br>FMA: Shoulder/Elbow/Forearm: BRT↑; Wrist: BRT↑; Hand: CT↑; Coordination: -; total: BRT↑;<br>RMS: SF (first 30% phase): -; SF (late 30% phase): -; WE (first 30% phase): BRT↑; WE (late 30% phase): -; WF (first	RTBP demonstrated superior upper limb motor and task performance outcomes compared to therapists-facilitated task training when both were preceded by a 10-minute sensorimotor stimulation session.

								30% phase): -; WF (late 30% phase): -;	
**Hesse et al. (2005)**	4 to 8 weeks after stroke causing severe arm paresis (FMA score <18)	BRT (n=22): BRT (20 min/session) + PT (45 min/session) + OT (30 min/session)<br>BRT: Within 1 session, each patient practiced 200 of the elbow and 200 of the wrist cycles, totaling 400 cycles, or 800 repetitions, half in mode 1 and half in mode 2. Additionally, the patients could practice 25 to 50 repetitions in mode 3	Bi-Manu-Track<br><br>End-effector	mode 1 (passive–passive mode)<br>mode 2 (active–passive mode)<br>mode 3 (active–active mode)	forearm pronation-supination andwrist flexion-extension patterns	20 min/session, 5 sessions/week, for 6 weeks	CT (n=22): electrical muscle stimulation (20 min/session) + PT (45 min/session) + OT (30 min/session)<br>CT: Four- to 7-s trains of monophasic exponential pulses (75 Hz; 0.5 ms; 0 to 80 mA) were applied by 2 self-adhesive flexible electrodes (2.5×3 cm). The intensity was set to produce maximum wrist extension. Patients performed 60 to 80 wrist extensions per session, with an interstimulus interval between 8 and 15 s	FMA: total: BRT↑; FMA-proximal, FMA-distal: -;	The computerized active arm training produced a superior improvement in upper limb motor control and power compared with ES in severely affected stroke patients. This is probably attributable to the greater number of repetitions and the bilateral approach.
**Yang et al. (2012)**	post-stroke; mild-to-moderate motor impairment	BRT (n=7): BRT (75-80 min/session) + functional task practice (15-20 min/session) + tone normalization for the arm at the beginning	Bi-Manu-Track<br><br>End-effector	mode 1 (passive–passive mode)<br>mode 2 (active–passive mode)	forearm pronation-supination andwrist flexion-extension patterns	90-105 min/session, 5 sessions/week, for 4 weeks	URT (n=7): URT (75-80 min/session) + functional task practice (15-20 min/session) + tone normalization for the arm at the beginning	FMA: total: URT superior than BRT and CT; Proximal: URT superior than BRT and CT; Distal: -;<br>MRC score: total: -; Proximal: BRT superior than CT and URT, CT	The pilot study indicated that the URTP and BRTP might have differential benefits for movement improvement. URTP might be a more compelling approach to

	and end of therapy, if necessary (5 min) BRT: included 300–400 repetitions in Modes 1 and 2 of the elbow and wrist cycles and each movement with both arms restrained on the device	mode 3 (active–active mode)		and end of therapy, if necessary (5 min) URT: same mode as BRT, but only use paretic arm to practice. CT (n=7): The therapeutic activities in the control group involved weight bearing, stretching, strengthening of the paretic arm, coordination tasks, unilateral and bilateral fine motor tasks, and balance.	superior than URT; Distal: -; Grip strength: bilateral condition: URT superior than BRT and CT; unilateral condition: -; MAS score: -	improving upper-limb motor impairment, muscle power, and strength at the distal joints than BRTP, whereas BRTP could be an optimal approach to improving proximal muscle power.

ADL, activities of daily living; AOU, amount of use; BAT, Bilateral assisted training ; BBT, Box and Block Test; BHT, Bilateral hybrid training; BRT, Bilateral robot-assisted training; CAHAI, Chedoke Arm and Hand Activity Inventory; CT, conventional training; FAS, functional ability scores; FMA, Fugl-Meyer Assessment Scale; FIM, Functional Independence Measure; GAS, goal attainment scaling; MAL, Motor Activity Log; MAS, Modified Ashworth Scale; mCIT, modified constraint-induced therapy; mRS, modified Rankin Scale; NEADL, Nottingham Extended Activities of Daily Living; QOM, quality of movement; SIS, Stroke Impact Scale; URT, Unilateral robot-assisted training; WMFT, Wolf Motor Function Test.

Supplementary Table S2. | Specific description of intervention methods.

Tier	Exercise type	Exercise names and description	Physiological/anatomical target	Target duration per session	Target intensity	Schedule	Criteria for moving to next tier
Tier1	Aircraft Wars	Patients perform shoulder abduction activities in the horizontal plane, moving left and right to fire bullets, with the difficulty of the training increasing as the patient completes the task.	increase shoulder abduction mobility and the patient's coordination, control and flexibility when performing abduction activities.	10min	1. passive activity when the patient's muscles are in a small amount of contraction or no contraction, 2. active or resistance movement when the patient has some joint movement in the affected upper limb after the stroke but the movement is very slight.	6 times a week, once a day, for a total of 3 weeks	Complete the current exercise without difficulties in execution of movements and adverse effects
	Shooting Mosquitoes	Mosquitoes and butterflies will appear on the screen from time to time and the patient will need to swat the mosquitoes and avoid the butterflies. 1 point will be added for each successful swat and 10 points will be deducted for hitting a butterfly.	increase shoulder pronation and abduction mobility, Elbow Flexion Mobility as well as coordination, control and flexibility of the patient's limbs when performing pronation and abduction activities.				
	Ocean Exploration	Patients complete the big fish eat small fish mission by moving their shoulders up and down for a total of 5 stars. When eaten by a big shark, it decreases by one heart, and as they eat more small fish, their body increases in size and they can have the power to eat big sharks.	increase shoulder pronation mobility, as well as coordination, control and flexibility of the patient's limbs when performing pronation activities.				

Tier2	Drumming	The patient performs up and down drumming by perform symmetric movements on the paretic side limb using the motor information of the non-paretic side limb	increase shoulder pronation mobility, as well as coordination, control and flexibility of the patient's limbs when performing pronation activities.	5min rounds, patient repeats 6 rounds	1. passive activity when the patient's muscles are in a small amount of contraction or no contraction, 2. active or resistance movement when the patient has some joint movement in the affected upper limb after the stroke but the movement is very slight.	6 times a week, once a day, for a total of 3 weeks	Complete the current exercise without difficulties in execution of movements and adverse effects
	Flagging	The patient waves the flag from side to side by perform symmetric movements on the paretic side limb using the motor information of the non-paretic side limb	increase shoulder abduction mobility and the patient's coordination, control and flexibility when performing abduction activities.				
	Paddle	The patient performs a side-to-side paddling motion by perform symmetric movements on the paretic side limb using the motor information of the non-paretic side limb	increase shoulder pronation and abduction mobility, Elbow Flexion Mobility as well as coordination, control and flexibility of the patient's limbs when performing pronation and abduction activities.				
Tier3	Physiotherapy	1.Passive movements from the proximal to the distal joints in the pain-free or less painful range, elbow extension and forward flexion of the shoulder with the help of the healthy hand through the Bobath grip<br>2.For patients who have some motor function in the upper limbs and who have reasonable muscle strength, the aim is to strengthen the extensor muscles and suppress flexor tone, and to strengthen motor control and coordination training for the hemiplegic upper	to promote the recovery of muscle strength and the emergence of active activities through active and passive training, to strengthen coordination and selective random movements and to intensify training in activities of daily living.	30min	1. passive activity when the patient's muscles are in a small amount of contraction or no contraction, 2. active or resistance movement when the patient has some joint movement in the affected upper limb after the stroke but the movement is very slight.	6 times a week, once a day, for a total of 3 weeks	Complete the current exercise without difficulties in execution of movements and adverse effects

		limbs according to their characteristics.					
	Occupational therapy	1.The functional tasks included reaching to move a cup, grasping and releasing blocks, picking up coins, barrel rolling training, wiping a table with two hands, pegging board.<br>2. Daily living training includes dressing, grooming, drinking, eating, etc.	to strengthen coordination and selective random movements and to intensify training in activities of daily living.	30min			
	Physical factor therapy	Functional electrical stimulation.	Increasing muscle contraction in the upper extremity	30min	Patient tolerance intensity		

Supplementary Table S3. | Missing data patterns for outcome variables (1= complete, 0 = missing).

	CT Group			URT Group			BRT Group		
	Frequency	Pattern		Frequency	Pattern		Frequency	Pattern	
		Baseline	Post intervention		Baseline	Post intervention		Baseline	Post intervention
sEMG parameters in both four muscle parts	24	1	1	21	1	1	24	1	1
				2	0	0			
MEP	23	1	1	22	1	1	24	1	1
	1	0	0	1	0	0			

sEMG, Surface electromyography; MEP, Motor Evoked Potential.

Supplementary Table S4. | Post hoc test results.

	Pairwise comparison of estimates of treatment effects (P value*)		
	Control vs. URT	Control vs. BRT	URT vs. BRT
**Primary outcome**			
FMA-UE	0.504	0.0043	0.0159
**Secondary outcomes**			
MBI	0.512	0.0055	0.0091
RMS biceps	0.788	0.592	0.452
RMS anterior deltoid bundle	0.605	0.0011	0.0034
RMS middle deltoid	0.230	0.129	0.864
RMS triceps brachii bundle	0.466	0.416	0.933
iEMG biceps	0.843	0.691	0.583
iEMG anterior deltoid bundle	0.908	0.0006	0.001
iEMG middle deltoid	0.200	0.173	0.956
iEMG triceps brachii bundle	0.482	0.396	0.885

*Post-hoc comparisons were based on t-test using Bonferroni correction for 3 tests, p-values of < 0.0167 were considered statistically significant.

BRT, bilateral robotic training; FMA-UE, Fugl-Meyer Assessment-Upper Extremities; iEMG, integrated electromyographic; MBI, Modified Barthel Index; RMS, root mean square; URT, unilateral robotic training

References

HESSE, S., WERNER, C., POHL, M., RUECKRIEM, S., MEHRHOLZ, J. & LINGNAU, M. L. 2005. Computerized arm training improves the motor control of the severely affected arm after stroke: a single-blinded randomized trial in two centers. *Stroke,* 36**,** 1960-6.

HSIEH, Y. W., LIING, R. J., LIN, K. C., WU, C. Y., LIOU, T. H., LIN, J. C. & HUNG, J. W. 2016. Sequencing bilateral robot-assisted arm therapy and constraint-induced therapy improves reach to press and trunk kinematics in patients with stroke. *J Neuroeng Rehabil,* 13**,** 31.

HSIEH, Y. W., WU, C. Y., WANG, W. E., LIN, K. C., CHANG, K. C., CHEN, C. C. & LIU, C. T. 2017. Bilateral robotic priming before task-oriented approach in subacute stroke rehabilitation: a pilot randomized controlled trial. *Clin Rehabil,* 31**,** 225-233.

HSU, H. Y., CHIU, H. Y., KUAN, T. S., TSAI, C. L., SU, F. C. & KUO, L. C. 2019. Robotic-assisted therapy with bilateral practice improves task and motor performance in the upper extremities of chronic stroke patients: A randomised controlled trial. *Aust Occup Ther J,* 66**,** 637-647.

HUNG, C. S., HSIEH, Y. W., WU, C. Y., CHEN, Y. J., LIN, K. C., CHEN, C. L., YAO, K. G., LIU, C. T. & HORNG, Y. S. 2019a. Hybrid Rehabilitation Therapies on Upper-Limb Function and Goal Attainment in Chronic Stroke. *OTJR (Thorofare N J),* 39**,** 116-123.

HUNG, C. S., LIN, K. C., CHANG, W. Y., HUANG, W. C., CHANG, Y. J., CHEN, C. L., GRACE YAO, K. & LEE, Y. Y. 2019b. Unilateral vs Bilateral Hybrid Approaches for Upper Limb Rehabilitation in Chronic Stroke: A Randomized Controlled Trial. *Arch Phys Med Rehabil,* 100**,** 2225-2232.

LIAO, W. W., WU, C. Y., HSIEH, Y. W., LIN, K. C. & CHANG, W. Y. 2012. Effects of robot-assisted upper limb rehabilitation on daily function and real-world arm activity in patients with chronic stroke: a randomized controlled trial. *Clin Rehabil,* 26**,** 111-20.

WU, C. Y., YANG, C. L., CHEN, M. D., LIN, K. C. & WU, L. L. 2013. Unilateral versus bilateral robot-assisted rehabilitation on arm-trunk control and functions post stroke: a randomized controlled trial. *J Neuroeng Rehabil,* 10**,** 35.

WU, C. Y., YANG, C. L., CHUANG, L. L., LIN, K. C., CHEN, H. C., CHEN, M. D. & HUANG, W. C. 2012. Effect of therapist-based versus robot-assisted bilateral arm training on motor control, functional performance, and quality of life after chronic stroke: a clinical trial. *Phys Ther,* 92**,** 1006-16.

YANG, C.-L., LIN, K.-C., CHEN, H.-C., WU, C.-Y. & CHEN, C.-L. 2012. Pilot comparative study of unilateral and bilateral robot-assisted training on upper-extremity performance in patients with stroke. *The American Journal of Occupational Therapy : Official Publication of the American Occupational Therapy Association,* 66**,** 198-206.
